# *N*-[4-(4-Nitro­phen­oxy)phen­yl]penta­n­amide

**DOI:** 10.1107/S1600536812036744

**Published:** 2012-08-31

**Authors:** Asifan Nigar, Zareen Akhter, Vickie McKee, Rizwan Hussain

**Affiliations:** a Department of Chemistry, Quaid-i-Azam University, Islamabad, Pakistan; b Chemistry Department, Loughborough University, Loughborough LE11 3TU, England; c NESCOM, PO Box 2216, Islamabad, Pakistan

## Abstract

The asymmetric unit of the title compound, C_17_H_18_N_2_O_4_, contains two independent mol­ecules (*A* and *B*) differing principally in the conformations of the alkyl chains, *anti* for molecule *A* and *gauche* for molecule *B*. The dihedral angles between the aromatic rings are 82.51 (6) and 82.25 (6)° in the two molecules. In the crystal, amide–amide inter­actions (as N—H⋯O=C) results in distinct chains of *A* and *B* mol­ecules running parallel to the *a*-axis direction. C—H⋯O inter­actions also occur.

## Related literature

For the related structures *N*-(4-(4-nitro­phen­oxy)phen­yl)pro­pionamide, 4-nitro-*N*-(4-(4-nitro­phen­oxy)phen­yl)benzamide and *N*-[4-(4-nitro­phen­oxy)phen­yl]acetamide see: Nigar *et al.* (2008 ▶), Butt *et al.* (2007 ▶) and Nigar *et al.* (2012 ▶), respectively. 
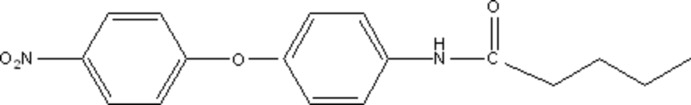


## Experimental

### 

#### Crystal data

C_17_H_18_N_2_O_4_
*M* *_r_* = 314.33
Triclinic, 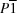

*a* = 4.9776 (5) Å
*b* = 10.1139 (10) Å
*c* = 30.572 (3) Å
α = 92.069 (2)°
β = 90.087 (2)°
γ = 91.060 (2)°
*V* = 1537.8 (3) Å^3^
*Z* = 4
Mo *K*α radiation
μ = 0.10 mm^−1^
*T* = 150 K
0.33 × 0.32 × 0.13 mm

#### Data collection

Bruker APEXII CCD diffractometer
Absorption correction: multi-scan (*SADABS*; Sheldrick, 2008*a* ▶) *T*_min_ = 0.968, *T*_max_ = 0.987
17614 measured reflections
6018 independent reflections
4664 reflections with *I* > 2σ(*I*)
*R*_int_ = 0.033

#### Refinement

*R*[*F* ^2^ > 2σ(*F* ^2^)] = 0.056
*wR*(*F* ^2^) = 0.135
*S* = 1.09
6018 reflections
415 parameters
H-atom parameters constrained
Δρ_max_ = 0.34 e Å^−3^
Δρ_min_ = −0.21 e Å^−3^

### 

Data collection: *APEX2* (Bruker 1998 ▶); cell refinement: *SAINT* (Bruker 1998 ▶); data reduction: *SAINT*; program(s) used to solve structure: *SHELXS97* (Sheldrick, 2008*b* ▶); program(s) used to refine structure: *SHELXL97* (Sheldrick, 2008*b* ▶); molecular graphics: *SHELXTL* (Sheldrick, 2008*b* ▶) and *Mercury* (Macrae *et al.*, 2008 ▶); software used to prepare material for publication: *SHELXTL*.

## Supplementary Material

Crystal structure: contains datablock(s) I, global. DOI: 10.1107/S1600536812036744/gg2093sup1.cif

Structure factors: contains datablock(s) I. DOI: 10.1107/S1600536812036744/gg2093Isup2.hkl

Supplementary material file. DOI: 10.1107/S1600536812036744/gg2093Isup3.cml

Additional supplementary materials:  crystallographic information; 3D view; checkCIF report

## Figures and Tables

**Table 1:** Hydrogen-bond geometry (Å, °)

*D*—H⋯*A*	*D*—H	H⋯*A*	*D*⋯*A*	*D*—H⋯*A*
N2*A*—H2*A*1⋯O4*A* ^i^	0.88	2.08	2.955 (2)	178
N2*B*—H2*B*1⋯O4*B* ^i^	0.88	2.05	2.929 (2)	174
C3*A*—H3*A*⋯O1*A* ^ii^	0.95	2.49	3.341 (3)	150
C5*A*—H5*A*⋯O2*A* ^iii^	0.95	2.62	3.376 (3)	137
C3*B*—H3*B*⋯O1*B* ^iv^	0.95	2.49	3.360 (3)	153

Symmetry codes: (i) 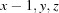; (ii) 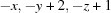; (iii) 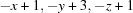; (iv) 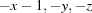.

**Fig. 1.**
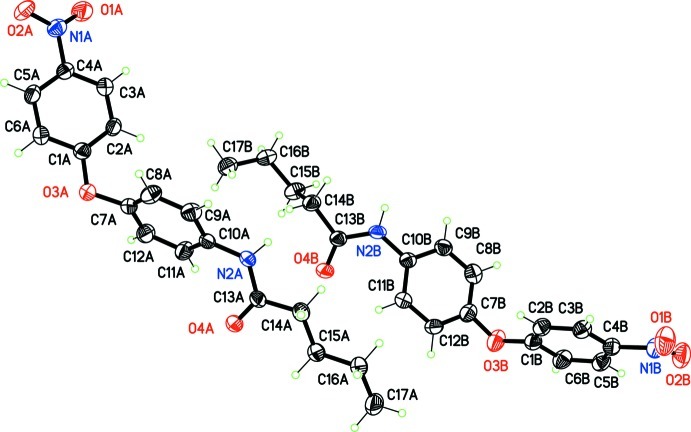
Perspective view of the two independent molecules in the asymmetric unit. Thermal ellipsoids shown at the 50% probability level.

**Fig. 2.**
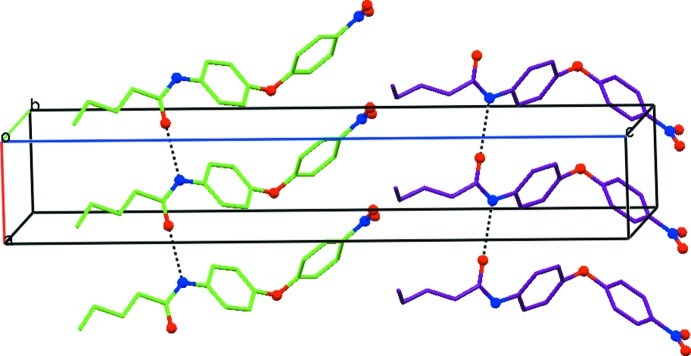
Packing diagram showing the NH ··· O ═C H-bonds. Carbon atoms coloured according to symmetry equivalence.

**Fig. 3.**
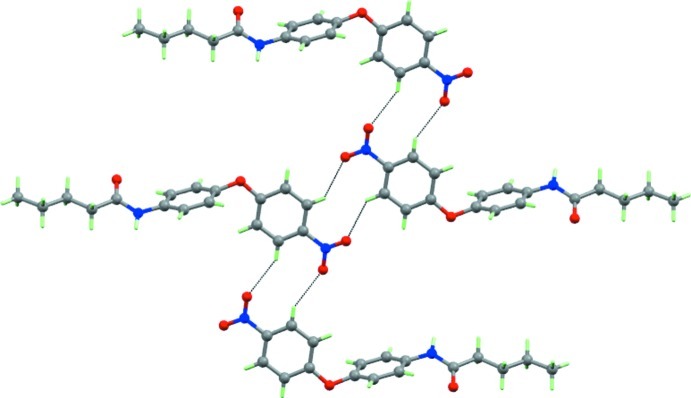
C—H···O interactions between 'A' molecules.

**Fig. 4.**
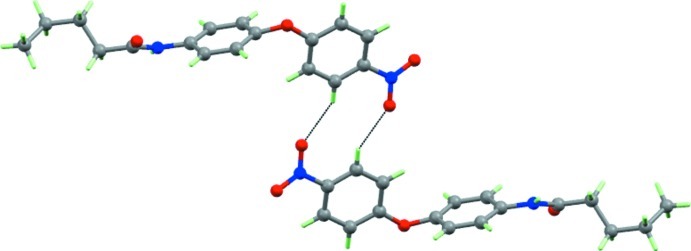
C—H···O interactions between 'B' molecules.

## supplementary crystallographic information

### Comment

The crystal structure of *N*-[4-(4-Nitrophenoxy)phenyl]acetamide (Nigar *et al.*2012) has been published and is related to that of the title compound, (Fig. 1).

There are two independent molecules in the asymmetric unit, differing primarily in the conformations of the alkyl chains (Fig. 1). The unsaturated sections of the molecules have quite similar orientations, with interplanar angles between the mean planes of the two aromatic rings of 82.51 (6)° for the molecule with 'A' labels and 82.25 (6)° for the molecule with 'B' labels.

Each molecule is linked to two crystallographically identical molecules *via* H-bonding involving the amide groups (N—H ···O═C distances 2.956 (2) Å and 2.929 (2) Å for N2A···O4A and N2B···O4B respectively, both under symmetry operation *x* - 1, *y*, *z*). This results in separate H-bonded chains of 'A' and 'B' molecules running parallel to the *a* axis (Fig 2, Table 3).

The nitro groups are involved in weaker H-bonding to aromatic C—H groups. Each molecule 'A' makes four bonds with identical neighbours, linking the chains together (C3A—H···O1A 3.341 (3) Å under -*x*, 2 - *y*, 1 - *z* and C5A—H···O2A 3.376 (3) Å under 1 - *x*, 3 - *y*, 1 - *z*, Fig 3, Table 3). Molecule 'B' only forms one such interaction; C3B···O1B 3.360 (3) Å under -1 - *x*,-*y*,-*z* (Fig 4, Table 3).

### Experimental

Synthesis of*N*-[4-(4'-Nitrophenoxy)phenyl] pentanamide

A mixture of 2.50 g (25 mmol) 4-aminophenol, 3.46 g (25 mmol) anhydrous K_2_CO_3_ and 2.65 ml (25 mmol) 4-nitrofluorobenzene in 35 ml DMF was heated at 373 K for 18 h in an inert atmosphere. After cooling to room temperature, the reaction mixture was poured into 400 ml of water to yield a yellow solid. The product was filtered, dried, and then recrystallized from n-hexane (yield, 86%). In the second step, pentanoic acid and thionylchloride were refluxed in equimolar amounts for 30 min. The excessive amount of thionylchloride was rotary evaporated and pentanoylchloride obtained was reacted with 4-(4-nitrophenoxy) aniline, as prepared in the first step, in appropriate molar ratios. THF was used as solvent and 1 ml of triethylamine was also added for 1.0 g of 4-(4-nitrophenoxy) aniline. The reaction mixture was refluxed for 2 h under inert conditions and allowed to stand overnight at room temperature. The settled salt was filtered off and filtrate was evaporated to get the crude product, which was recrystallized from toluene (89% yield, m.p. 390 K)

### Refinement

C-bound H atoms were positioned geometrically in ideal distances (0.95 Å for aromatic H and 0.98, 0.99 Å for aliphatic H) and treated as riding on their parent atoms. N-Bound atoms were positioned in a similar fashion at 0.88 Å.

### Crystal data

**Table:**

C_17_H_18_N_2_O_4_	*Z* = 4
*M**_r_* = 314.33	*F*(000) = 664
Triclinic, *P*1	*D*_x_ = 1.358 Mg m^−^^3^
Hall symbol: -P 1	Melting point: 390 K
*a* = 4.9776 (5) Å	Mo *K*α radiation, λ = 0.71073 Å
*b* = 10.1139 (10) Å	Cell parameters from 4085 reflections
*c* = 30.572 (3) Å	θ = 2.4–25.3°
α = 92.069 (2)°	µ = 0.10 mm^−^^1^
β = 90.087 (2)°	*T* = 150 K
γ = 91.060 (2)°	Wedge, brown
*V* = 1537.8 (3) Å^3^	0.33 × 0.32 × 0.13 mm

### Data collection

**Table:**

Bruker APEXII CCD diffractometer	6018 independent reflections
Radiation source: fine-focus sealed tube	4664 reflections with *I* > 2σ(*I*)
Graphite monochromator	*R*_int_ = 0.033
φ and ω scans	θ_max_ = 26.0°, θ_min_ = 0.7°
Absorption correction: multi-scan (*SADABS*; Sheldrick, 2008*a*)	*h* = −6→6
*T*_min_ = 0.968, *T*_max_ = 0.987	*k* = −12→12
17614 measured reflections	*l* = −37→37

### Refinement

**Table:**

Refinement on *F*^2^	Primary atom site location: structure-invariant direct methods
Least-squares matrix: full	Secondary atom site location: difference Fourier map
*R*[*F*^2^ > 2σ(*F*^2^)] = 0.056	Hydrogen site location: inferred from neighbouring sites
*wR*(*F*^2^) = 0.135	H-atom parameters constrained
*S* = 1.09	*w* = 1/[σ^2^(*F*_o_^2^) + (0.0389*P*)^2^ + 1.2455*P*] where *P* = (*F*_o_^2^ + 2*F*_c_^2^)/3
6018 reflections	(Δ/σ)_max_ < 0.001
415 parameters	Δρ_max_ = 0.34 e Å^−^^3^
0 restraints	Δρ_min_ = −0.21 e Å^−^^3^

### Special details

**Table:**

Geometry. All e.s.d.'s (except the e.s.d. in the dihedral angle between two l.s. planes) are estimated using the full covariance matrix. The cell e.s.d.'s are taken into account individually in the estimation of e.s.d.'s in distances, angles and torsion angles; correlations between e.s.d.'s in cell parameters are only used when they are defined by crystal symmetry. An approximate (isotropic) treatment of cell e.s.d.'s is used for estimating e.s.d.'s involving l.s. planes.
Refinement. Refinement of *F*^2^ against ALL reflections. The weighted *R*-factor *wR* and goodness of fit *S* are based on *F*^2^, conventional *R*-factors *R* are based on *F*, with *F* set to zero for negative *F*^2^. The threshold expression of *F*^2^ > σ(*F*^2^) is used only for calculating *R*-factors(gt) *etc*. and is not relevant to the choice of reflections for refinement. *R*-factors based on *F*^2^ are statistically about twice as large as those based on *F*, and *R*- factors based on ALL data will be even larger.

### Fractional atomic coordinates and isotropic or equivalent isotropic displacement parameters (Å^2^)

**Table:**

	*x*	*y*	*z*	*U*_iso_*/*U*_eq_	
C1A	0.6952 (5)	1.1513 (2)	0.41515 (8)	0.0331 (5)	
C2A	0.5007 (5)	1.0629 (2)	0.42860 (8)	0.0323 (5)	
H2A	0.4789	0.9789	0.4140	0.039*	
C3A	0.3375 (5)	1.0977 (2)	0.46368 (8)	0.0330 (5)	
H3A	0.2037	1.0377	0.4736	0.040*	
C4A	0.3722 (5)	1.2207 (2)	0.48397 (7)	0.0313 (5)	
N1A	0.1898 (4)	1.2606 (2)	0.51924 (7)	0.0387 (5)	
O1A	0.0553 (4)	1.17409 (19)	0.53686 (6)	0.0511 (5)	
O2A	0.1772 (4)	1.37879 (18)	0.52958 (6)	0.0489 (5)	
C5A	0.5702 (5)	1.3092 (2)	0.47140 (8)	0.0350 (5)	
H5A	0.5933	1.3926	0.4864	0.042*	
C6A	0.7335 (5)	1.2738 (2)	0.43667 (8)	0.0357 (5)	
H6A	0.8713	1.3327	0.4275	0.043*	
O3A	0.8658 (3)	1.12568 (18)	0.38088 (6)	0.0442 (5)	
C7A	0.7902 (5)	1.0268 (2)	0.34970 (8)	0.0349 (5)	
C8A	0.5830 (5)	1.0467 (2)	0.32109 (8)	0.0394 (6)	
H8A	0.4787	1.1242	0.3240	0.047*	
C9A	0.5272 (5)	0.9532 (2)	0.28806 (8)	0.0360 (6)	
H9A	0.3832	0.9663	0.2683	0.043*	
C10A	0.6800 (4)	0.8405 (2)	0.28355 (7)	0.0278 (5)	
C11A	0.8841 (5)	0.8202 (2)	0.31316 (8)	0.0377 (6)	
H11A	0.9872	0.7422	0.3107	0.045*	
C12A	0.9385 (5)	0.9137 (3)	0.34648 (8)	0.0417 (6)	
H12A	1.0777	0.8996	0.3670	0.050*	
N2A	0.6189 (4)	0.74848 (19)	0.24853 (6)	0.0305 (4)	
H2A1	0.4481	0.7321	0.2427	0.037*	
C13A	0.8018 (4)	0.6841 (2)	0.22349 (7)	0.0295 (5)	
O4A	1.0444 (3)	0.68820 (17)	0.23109 (5)	0.0367 (4)	
C14A	0.6836 (5)	0.6108 (3)	0.18369 (8)	0.0380 (6)	
H14A	0.5192	0.5624	0.1926	0.046*	
H14B	0.6300	0.6763	0.1622	0.046*	
C15A	0.8712 (5)	0.5141 (3)	0.16170 (8)	0.0371 (6)	
H15A	0.9136	0.4442	0.1823	0.044*	
H15B	1.0413	0.5609	0.1546	0.044*	
C16A	0.7546 (5)	0.4497 (3)	0.11993 (8)	0.0393 (6)	
H16A	0.7300	0.5180	0.0980	0.047*	
H16B	0.5762	0.4100	0.1262	0.047*	
C17A	0.9380 (6)	0.3430 (3)	0.10128 (10)	0.0523 (7)	
H17A	0.8580	0.3032	0.0745	0.078*	
H17B	0.9603	0.2746	0.1228	0.078*	
H17C	1.1137	0.3824	0.0945	0.078*	
C1B	0.1775 (5)	−0.1421 (2)	0.09199 (8)	0.0355 (5)	
C2B	0.0018 (5)	−0.0524 (2)	0.07505 (8)	0.0355 (5)	
H2B	−0.0041	0.0355	0.0870	0.043*	
C3B	−0.1649 (5)	−0.0917 (2)	0.04060 (8)	0.0367 (6)	
H3B	−0.2868	−0.0314	0.0287	0.044*	
C4B	−0.1514 (5)	−0.2198 (2)	0.02386 (8)	0.0358 (5)	
N1B	−0.3357 (5)	−0.2628 (2)	−0.01144 (7)	0.0452 (5)	
O1B	−0.4575 (5)	−0.1779 (2)	−0.03068 (7)	0.0630 (6)	
O2B	−0.3609 (4)	−0.3815 (2)	−0.02012 (7)	0.0585 (6)	
C5B	0.0274 (5)	−0.3092 (2)	0.03978 (8)	0.0403 (6)	
H5B	0.0347	−0.3966	0.0274	0.048*	
C6B	0.1945 (5)	−0.2697 (3)	0.07381 (9)	0.0417 (6)	
H6B	0.3210	−0.3293	0.0849	0.050*	
O3B	0.3504 (4)	−0.11092 (19)	0.12585 (6)	0.0511 (5)	
C7B	0.2903 (5)	−0.0041 (2)	0.15468 (8)	0.0374 (6)	
C8B	0.0770 (5)	−0.0121 (3)	0.18294 (8)	0.0414 (6)	
H8B	−0.0434	−0.0861	0.1812	0.050*	
C9B	0.0393 (5)	0.0892 (2)	0.21400 (8)	0.0364 (6)	
H9B	−0.1091	0.0848	0.2334	0.044*	
C10B	0.2158 (4)	0.1966 (2)	0.21698 (7)	0.0292 (5)	
C11B	0.4253 (5)	0.2041 (3)	0.18721 (8)	0.0383 (6)	
H11B	0.5443	0.2787	0.1883	0.046*	
C12B	0.4618 (5)	0.1036 (3)	0.15602 (8)	0.0429 (6)	
H12B	0.6050	0.1092	0.1356	0.051*	
N2B	0.1717 (4)	0.29683 (19)	0.24937 (6)	0.0318 (4)	
H2B1	0.0035	0.3139	0.2560	0.038*	
C13B	0.3637 (4)	0.3693 (2)	0.27129 (7)	0.0313 (5)	
O4B	0.6048 (3)	0.35562 (18)	0.26473 (6)	0.0424 (4)	
C14B	0.2633 (5)	0.4669 (2)	0.30584 (8)	0.0359 (6)	
H14C	0.0654	0.4723	0.3036	0.043*	
H14D	0.3410	0.5557	0.3006	0.043*	
C15B	0.3385 (5)	0.4267 (3)	0.35172 (8)	0.0417 (6)	
H15C	0.5355	0.4158	0.3533	0.050*	
H15D	0.2522	0.3401	0.3575	0.050*	
C16B	0.2540 (5)	0.5274 (3)	0.38710 (8)	0.0402 (6)	
H16C	0.0604	0.5449	0.3834	0.048*	
H16D	0.2775	0.4882	0.4160	0.048*	
C17B	0.4077 (5)	0.6579 (3)	0.38696 (9)	0.0447 (6)	
H17D	0.3405	0.7170	0.4104	0.067*	
H17E	0.3830	0.6988	0.3587	0.067*	
H17F	0.5992	0.6424	0.3917	0.067*	

### Atomic displacement parameters (Å^2^)

**Table:**

	*U*^11^	*U*^22^	*U*^33^	*U*^12^	*U*^13^	*U*^23^
C1A	0.0290 (12)	0.0373 (13)	0.0328 (12)	0.0016 (10)	−0.0005 (10)	−0.0023 (10)
C2A	0.0350 (13)	0.0281 (12)	0.0333 (13)	0.0004 (10)	−0.0023 (10)	−0.0051 (10)
C3A	0.0340 (12)	0.0312 (12)	0.0338 (13)	−0.0013 (10)	−0.0010 (10)	0.0006 (10)
C4A	0.0317 (12)	0.0334 (12)	0.0287 (12)	0.0056 (10)	−0.0030 (9)	−0.0021 (9)
N1A	0.0404 (12)	0.0429 (13)	0.0325 (11)	0.0060 (10)	−0.0006 (9)	−0.0043 (9)
O1A	0.0585 (12)	0.0512 (12)	0.0433 (11)	−0.0046 (10)	0.0158 (9)	−0.0016 (9)
O2A	0.0591 (12)	0.0395 (11)	0.0473 (11)	0.0092 (9)	0.0072 (9)	−0.0122 (8)
C5A	0.0384 (13)	0.0292 (12)	0.0369 (13)	0.0012 (10)	−0.0039 (10)	−0.0056 (10)
C6A	0.0335 (13)	0.0324 (13)	0.0406 (14)	−0.0040 (10)	−0.0011 (10)	−0.0041 (10)
O3A	0.0358 (9)	0.0478 (11)	0.0471 (11)	−0.0099 (8)	0.0101 (8)	−0.0188 (8)
C7A	0.0294 (12)	0.0403 (14)	0.0340 (13)	−0.0053 (10)	0.0063 (10)	−0.0085 (10)
C8A	0.0432 (14)	0.0351 (13)	0.0399 (14)	0.0108 (11)	0.0033 (11)	−0.0010 (11)
C9A	0.0318 (12)	0.0428 (14)	0.0338 (13)	0.0086 (10)	−0.0025 (10)	0.0028 (11)
C10A	0.0234 (11)	0.0339 (12)	0.0260 (11)	−0.0025 (9)	0.0020 (9)	0.0001 (9)
C11A	0.0344 (13)	0.0391 (14)	0.0392 (14)	0.0100 (10)	−0.0067 (11)	−0.0070 (11)
C12A	0.0309 (13)	0.0523 (16)	0.0410 (14)	0.0042 (11)	−0.0082 (11)	−0.0110 (12)
N2A	0.0191 (9)	0.0385 (11)	0.0336 (10)	−0.0009 (8)	−0.0017 (8)	−0.0033 (8)
C13A	0.0227 (11)	0.0336 (12)	0.0321 (12)	−0.0008 (9)	−0.0020 (9)	0.0016 (10)
O4A	0.0223 (8)	0.0474 (10)	0.0398 (10)	0.0009 (7)	−0.0026 (7)	−0.0088 (8)
C14A	0.0256 (12)	0.0470 (15)	0.0408 (14)	0.0009 (10)	−0.0061 (10)	−0.0076 (11)
C15A	0.0294 (12)	0.0443 (14)	0.0371 (13)	−0.0013 (10)	−0.0028 (10)	−0.0035 (11)
C16A	0.0329 (13)	0.0474 (15)	0.0368 (14)	−0.0040 (11)	−0.0045 (10)	−0.0050 (11)
C17A	0.0418 (15)	0.0628 (19)	0.0507 (17)	0.0022 (13)	−0.0084 (13)	−0.0196 (14)
C1B	0.0318 (12)	0.0384 (14)	0.0361 (13)	0.0053 (10)	−0.0003 (10)	−0.0036 (10)
C2B	0.0369 (13)	0.0302 (12)	0.0391 (14)	0.0046 (10)	0.0012 (10)	−0.0042 (10)
C3B	0.0386 (13)	0.0372 (13)	0.0343 (13)	0.0054 (11)	0.0003 (10)	−0.0006 (10)
C4B	0.0372 (13)	0.0385 (14)	0.0312 (13)	−0.0057 (11)	0.0029 (10)	−0.0001 (10)
N1B	0.0473 (13)	0.0493 (14)	0.0383 (12)	−0.0052 (11)	−0.0008 (10)	−0.0044 (11)
O1B	0.0697 (14)	0.0609 (14)	0.0582 (13)	0.0056 (11)	−0.0255 (11)	−0.0036 (11)
O2B	0.0713 (14)	0.0471 (12)	0.0557 (13)	−0.0152 (10)	−0.0069 (10)	−0.0106 (10)
C5B	0.0498 (15)	0.0301 (13)	0.0405 (14)	0.0019 (11)	0.0043 (12)	−0.0044 (11)
C6B	0.0457 (15)	0.0366 (14)	0.0429 (15)	0.0117 (11)	−0.0014 (12)	−0.0033 (11)
O3B	0.0468 (11)	0.0528 (12)	0.0528 (12)	0.0211 (9)	−0.0167 (9)	−0.0187 (9)
C7B	0.0359 (13)	0.0403 (14)	0.0360 (13)	0.0123 (11)	−0.0075 (11)	−0.0065 (11)
C8B	0.0425 (15)	0.0413 (15)	0.0406 (14)	−0.0049 (11)	−0.0045 (11)	0.0050 (11)
C9B	0.0326 (13)	0.0442 (14)	0.0323 (13)	−0.0020 (11)	0.0025 (10)	0.0030 (11)
C10B	0.0245 (11)	0.0353 (12)	0.0280 (12)	0.0071 (9)	−0.0026 (9)	0.0021 (9)
C11B	0.0311 (13)	0.0444 (15)	0.0388 (14)	−0.0041 (11)	0.0050 (10)	−0.0057 (11)
C12B	0.0301 (13)	0.0611 (17)	0.0366 (14)	0.0025 (12)	0.0051 (10)	−0.0111 (12)
N2B	0.0216 (9)	0.0440 (12)	0.0297 (10)	0.0067 (8)	0.0025 (8)	−0.0014 (9)
C13B	0.0259 (12)	0.0376 (13)	0.0304 (12)	0.0052 (10)	−0.0011 (9)	−0.0004 (10)
O4B	0.0221 (8)	0.0595 (12)	0.0445 (10)	0.0046 (8)	0.0008 (7)	−0.0129 (9)
C14B	0.0298 (12)	0.0402 (14)	0.0378 (14)	0.0096 (10)	−0.0015 (10)	−0.0035 (11)
C15B	0.0467 (15)	0.0408 (14)	0.0382 (14)	0.0114 (12)	0.0052 (11)	0.0019 (11)
C16B	0.0427 (14)	0.0446 (15)	0.0337 (13)	0.0106 (11)	0.0067 (11)	−0.0003 (11)
C17B	0.0429 (15)	0.0489 (16)	0.0418 (15)	0.0079 (12)	−0.0005 (12)	−0.0061 (12)

### Geometric parameters (Å, º)

**Table:**

C1A—O3A	1.370 (3)	C1B—O3B	1.369 (3)
C1A—C2A	1.379 (3)	C1B—C2B	1.385 (3)
C1A—C6A	1.391 (3)	C1B—C6B	1.390 (3)
C2A—C3A	1.386 (3)	C2B—C3B	1.382 (3)
C2A—H2A	0.9500	C2B—H2B	0.9500
C3A—C4A	1.378 (3)	C3B—C4B	1.378 (3)
C3A—H3A	0.9500	C3B—H3B	0.9500
C4A—C5A	1.383 (3)	C4B—C5B	1.381 (3)
C4A—N1A	1.461 (3)	C4B—N1B	1.464 (3)
N1A—O2A	1.229 (3)	N1B—O2B	1.225 (3)
N1A—O1A	1.232 (3)	N1B—O1B	1.227 (3)
C5A—C6A	1.379 (3)	C5B—C6B	1.374 (4)
C5A—H5A	0.9500	C5B—H5B	0.9500
C6A—H6A	0.9500	C6B—H6B	0.9500
O3A—C7A	1.403 (3)	O3B—C7B	1.406 (3)
C7A—C12A	1.373 (3)	C7B—C12B	1.371 (4)
C7A—C8A	1.373 (3)	C7B—C8B	1.373 (4)
C8A—C9A	1.383 (3)	C8B—C9B	1.387 (4)
C8A—H8A	0.9500	C8B—H8B	0.9500
C9A—C10A	1.385 (3)	C9B—C10B	1.385 (3)
C9A—H9A	0.9500	C9B—H9B	0.9500
C10A—C11A	1.383 (3)	C10B—C11B	1.388 (3)
C10A—N2A	1.422 (3)	C10B—N2B	1.412 (3)
C11A—C12A	1.387 (3)	C11B—C12B	1.383 (3)
C11A—H11A	0.9500	C11B—H11B	0.9500
C12A—H12A	0.9500	C12B—H12B	0.9500
N2A—C13A	1.352 (3)	N2B—C13B	1.355 (3)
N2A—H2A1	0.8800	N2B—H2B1	0.8800
C13A—O4A	1.229 (3)	C13B—O4B	1.227 (3)
C13A—C14A	1.514 (3)	C13B—C14B	1.512 (3)
C14A—C15A	1.506 (3)	C14B—C15B	1.522 (3)
C14A—H14A	0.9900	C14B—H14C	0.9900
C14A—H14B	0.9900	C14B—H14D	0.9900
C15A—C16A	1.522 (3)	C15B—C16B	1.524 (3)
C15A—H15A	0.9900	C15B—H15C	0.9900
C15A—H15B	0.9900	C15B—H15D	0.9900
C16A—C17A	1.522 (4)	C16B—C17B	1.513 (4)
C16A—H16A	0.9900	C16B—H16C	0.9900
C16A—H16B	0.9900	C16B—H16D	0.9900
C17A—H17A	0.9800	C17B—H17D	0.9800
C17A—H17B	0.9800	C17B—H17E	0.9800
C17A—H17C	0.9800	C17B—H17F	0.9800
			
O3A—C1A—C2A	123.5 (2)	O3B—C1B—C2B	123.2 (2)
O3A—C1A—C6A	115.3 (2)	O3B—C1B—C6B	116.0 (2)
C2A—C1A—C6A	121.2 (2)	C2B—C1B—C6B	120.8 (2)
C1A—C2A—C3A	119.4 (2)	C3B—C2B—C1B	119.5 (2)
C1A—C2A—H2A	120.3	C3B—C2B—H2B	120.2
C3A—C2A—H2A	120.3	C1B—C2B—H2B	120.2
C4A—C3A—C2A	119.0 (2)	C4B—C3B—C2B	118.9 (2)
C4A—C3A—H3A	120.5	C4B—C3B—H3B	120.5
C2A—C3A—H3A	120.5	C2B—C3B—H3B	120.5
C3A—C4A—C5A	122.2 (2)	C3B—C4B—C5B	122.1 (2)
C3A—C4A—N1A	119.0 (2)	C3B—C4B—N1B	118.8 (2)
C5A—C4A—N1A	118.8 (2)	C5B—C4B—N1B	119.1 (2)
O2A—N1A—O1A	123.4 (2)	O2B—N1B—O1B	123.5 (2)
O2A—N1A—C4A	118.3 (2)	O2B—N1B—C4B	118.3 (2)
O1A—N1A—C4A	118.4 (2)	O1B—N1B—C4B	118.2 (2)
C6A—C5A—C4A	118.6 (2)	C6B—C5B—C4B	118.9 (2)
C6A—C5A—H5A	120.7	C6B—C5B—H5B	120.5
C4A—C5A—H5A	120.7	C4B—C5B—H5B	120.5
C5A—C6A—C1A	119.6 (2)	C5B—C6B—C1B	119.7 (2)
C5A—C6A—H6A	120.2	C5B—C6B—H6B	120.1
C1A—C6A—H6A	120.2	C1B—C6B—H6B	120.1
C1A—O3A—C7A	118.14 (18)	C1B—O3B—C7B	118.86 (18)
C12A—C7A—C8A	120.8 (2)	C12B—C7B—C8B	121.1 (2)
C12A—C7A—O3A	118.7 (2)	C12B—C7B—O3B	118.1 (2)
C8A—C7A—O3A	120.4 (2)	C8B—C7B—O3B	120.6 (2)
C7A—C8A—C9A	119.6 (2)	C7B—C8B—C9B	119.2 (2)
C7A—C8A—H8A	120.2	C7B—C8B—H8B	120.4
C9A—C8A—H8A	120.2	C9B—C8B—H8B	120.4
C8A—C9A—C10A	120.4 (2)	C10B—C9B—C8B	120.7 (2)
C8A—C9A—H9A	119.8	C10B—C9B—H9B	119.7
C10A—C9A—H9A	119.8	C8B—C9B—H9B	119.7
C11A—C10A—C9A	119.5 (2)	C9B—C10B—C11B	119.0 (2)
C11A—C10A—N2A	122.2 (2)	C9B—C10B—N2B	118.8 (2)
C9A—C10A—N2A	118.4 (2)	C11B—C10B—N2B	122.2 (2)
C10A—C11A—C12A	120.1 (2)	C12B—C11B—C10B	120.3 (2)
C10A—C11A—H11A	120.0	C12B—C11B—H11B	119.8
C12A—C11A—H11A	120.0	C10B—C11B—H11B	119.8
C7A—C12A—C11A	119.7 (2)	C7B—C12B—C11B	119.7 (2)
C7A—C12A—H12A	120.1	C7B—C12B—H12B	120.2
C11A—C12A—H12A	120.1	C11B—C12B—H12B	120.2
C13A—N2A—C10A	125.31 (18)	C13B—N2B—C10B	126.19 (18)
C13A—N2A—H2A1	117.3	C13B—N2B—H2B1	116.9
C10A—N2A—H2A1	117.3	C10B—N2B—H2B1	116.9
O4A—C13A—N2A	123.5 (2)	O4B—C13B—N2B	123.0 (2)
O4A—C13A—C14A	122.4 (2)	O4B—C13B—C14B	121.2 (2)
N2A—C13A—C14A	114.11 (19)	N2B—C13B—C14B	115.74 (19)
C15A—C14A—C13A	114.24 (19)	C13B—C14B—C15B	111.66 (19)
C15A—C14A—H14A	108.7	C13B—C14B—H14C	109.3
C13A—C14A—H14A	108.7	C15B—C14B—H14C	109.3
C15A—C14A—H14B	108.7	C13B—C14B—H14D	109.3
C13A—C14A—H14B	108.7	C15B—C14B—H14D	109.3
H14A—C14A—H14B	107.6	H14C—C14B—H14D	107.9
C14A—C15A—C16A	113.0 (2)	C14B—C15B—C16B	112.8 (2)
C14A—C15A—H15A	109.0	C14B—C15B—H15C	109.0
C16A—C15A—H15A	109.0	C16B—C15B—H15C	109.0
C14A—C15A—H15B	109.0	C14B—C15B—H15D	109.0
C16A—C15A—H15B	109.0	C16B—C15B—H15D	109.0
H15A—C15A—H15B	107.8	H15C—C15B—H15D	107.8
C15A—C16A—C17A	111.2 (2)	C17B—C16B—C15B	114.6 (2)
C15A—C16A—H16A	109.4	C17B—C16B—H16C	108.6
C17A—C16A—H16A	109.4	C15B—C16B—H16C	108.6
C15A—C16A—H16B	109.4	C17B—C16B—H16D	108.6
C17A—C16A—H16B	109.4	C15B—C16B—H16D	108.6
H16A—C16A—H16B	108.0	H16C—C16B—H16D	107.6
C16A—C17A—H17A	109.5	C16B—C17B—H17D	109.5
C16A—C17A—H17B	109.5	C16B—C17B—H17E	109.5
H17A—C17A—H17B	109.5	H17D—C17B—H17E	109.5
C16A—C17A—H17C	109.5	C16B—C17B—H17F	109.5
H17A—C17A—H17C	109.5	H17D—C17B—H17F	109.5
H17B—C17A—H17C	109.5	H17E—C17B—H17F	109.5

### Hydrogen-bond geometry (Å, º)

**Table:**

*D*—H···*A*	*D*—H	H···*A*	*D*···*A*	*D*—H···*A*
N2*A*—H2*A*1···O4*A*^i^	0.88	2.08	2.955 (2)	178
N2*B*—H2*B*1···O4*B*^i^	0.88	2.05	2.929 (2)	174
C3*A*—H3*A*···O1*A*^ii^	0.95	2.49	3.341 (3)	150
C5*A*—H5*A*···O2*A*^iii^	0.95	2.62	3.376 (3)	137
C3*B*—H3*B*···O1*B*^iv^	0.95	2.49	3.360 (3)	153

Symmetry codes: (i) *x*−1, *y*, *z*; (ii) −*x*, −*y*+2, −*z*+1; (iii) −*x*+1, −*y*+3, −*z*+1; (iv) −*x*−1, −*y*, −*z*.
